# Supplementary material for: Global fungal-host interactome mapping identifies host targets of candidalysin
Source: Nat Commun. 2024 Feb 27;15:1757. doi: 10.1038/s41467-024-46141-x (PMC10899660; doi:10.1038/s41467-024-46141-x)
Supplement: Supplementary file 1 — Supplementary Information [file 41467_2024_46141_MOESM1_ESM.pdf]

Supplementary information

Global fungal-host interactome mapping identifies host targets of candidalysin

Tian-Yi Zhang[1,*], Yao-Qi Chen[1,*], Jing-Cong Tan[1,*], Jin-An Zhou[1], Wan-Ning Chen[2], Tong Jiang[3], Jin-Yin Zha[4], Xiang-Kang Zeng, Bo-Wen Li[1], Lu-Qi Wei[1], Yun Zou, Lu-Yao Zhang, Yue-Mei Hong[1], Xiu-Li Wang[1], Run-Ze Zhu[1], Wan-Xing Xu[1], Jing Xi[1], Qin-Qin Wang[1], Lei Pan[5], Jian Zhang[4], Yang Luan[1], Rui-Xin Zhu[2], Hui Wang[1, #], Changbin Chen[3,#], Ning-Ning Liu[1,#]

Supplementary information includes:

Supplementary Tables: Supplementary Table 1-3.

Supplementary Figures: Supplementary Figure 1-6.

Supplementary Texts

Uncropped scans of source western blot gels related to supplementary figures

Supplementary Table 1. Key resources used in this study.

REAGENT/RESOURCE	SOURCE	IDENTIFIER
**Antibodies**		
**CCNH antibody**	CST	Cat#2927, RRID:AB_2259936
**gama-H_2AX antibody**	CST	Cat#9718S, RRID:AB_2118009
**Anti-gamma H2A.X (phospho S139) antibody**	Abcam	Cat#ab303656
**GAPDH (D16H11) XP® Rabbit mAb**	CST	Cat#5174S, RRID:AB_10622025
**Anti-Cyclin H/p34 antibody**	Abcam	Cat#EPR3929
**Anti-CDK2 (phospho T160) + CDK1 (phospho T161) antibody**	Abcam	Cat#EPR17621
**Mouse IgG (Magnetic Bead Conjugate)**	CST	Cat#5873
**DYKDDDDK Tag (D6W5B) Rabbit mAb**	CST	Cat#14793
**GFP (5G4) Mouse mAb (Magnetic Bead Conjugate)**	CST	Cat#67090
**GFP (4B10) Mouse mAb**	CST	Cat #2955
**Anti-rabbit lgG, HRP-linked Antibody**	CST	Cat#7074, RRID:AB_2099233
**Anti-mouse IgG, HRP-linked Antibody**	CST	Cat#7076
**Goat Anti-Mouse IgG H&L (Alexa Fluor® 647)**	Abcam	Cat#ab150115, RRID:AB_2687948
**Goat Anti-Rabbit IgG H&L (Alexa Fluor® 488)**	Abcam	Cat#ab150077
**Experimental models: organisms/strains**		
***Candida albicans* SC5314**	This study	N/A
**M1477 (BWP17+Clp30)**	Moyes et al., 2016	N/A
**M2057 (*ece1 Δ/Δ*)**	Moyes et al., 2016	N/A
**M2059 (*ece1Δ/Δ+ECE1*)**	Moyes et al., 2016	N/A
**M2174 (*ece1Δ/Δ+ECE1*$_{\Delta 184\text{-}279}$)**	Moyes et al., 2016	N/A
**Mice: BALB/c**	LINGCHANG BIOTECH	Permit Number: A2020071
**Flies: W1118**	Bloomington Stock Center	Cat#BL5905
**Chemicals, Peptides, and Recombinant Proteins**		

**Peptone**	Sigma- Aldrich	Cat# 39396
**Yeast extract**	BD	Cat# 210933
**Agar**	Sigma- Aldrich	Cat# A1296
**D-(+)- Glucose**	Sigma- Aldrich	Cat# G8270
**Yeast nitrogen base**	Sigma- Aldrich,	Cat# 51483
**Alanine, arginine, asparagine, aspartic acid, cysteine, glutamic acid, glutamine, glycine, isoleucine, lysine, methionine, phenylalanine, proline, serine, threonine, tyrosine, valine**	Sigma- Aldrich	N/A
**Calcein/PI Cell Viability/Cytotoxicity Assay Kit**	DOJINDO	Cat#C542
**Ammonium sulfate**	Sigma- Aldrich	Cat# A4418
**Adenine**	Sigma- Aldrich	Cat# A3159
**Uracil**	Sigma- Aldrich	Cat# U0750
**Histidine**	Sigma- Aldrich	Cat# H8000
**3- Amino-1, 2, 4- triazole**	Sigma- Aldrich	Cat# A8056
**Sodium hydroxide**	Macklin	Cat# S817968
**Glycerin**	Macklin	Cat# G810575
**Cycloheximide**	Sigma- Aldrich	Cat# C7698
**Platinum Taq DNA Polymerase High Fidelity**	Thermo Scientific™	Cat# 11304011
**6-well plates**	Thermo Scientific™	Cat#140675
**96-well plate**	Corning	Cat# 3788
**24 mm Transwell® cell culture plates**	Corning	Cat# 3450
**Lipofectamine™ 3000**	Thermo Scientific™	Cat#L3000001
**Aluminum film**	Bio-rad	Cat# MSF1001
**PCR plate sealing film**	Bio-rad	Cat# MSB1001
**0.22 µm filter**	Millipore	Cat# SLGV033RB
**15 cm Petri dish**	Corning	Cat# 430599
**Minimum Essential Medium, MEM**	Gibco	Cat# 11095098
**Fetal Bovine Serum, FBS**	Gibco	Cat# 10099-141C
**Penicillin/streptomycin**	Gibco	Cat# 15140122
**Trypsin-EDTA (0.25%), phenol red**	Gibco	Cat# 25200056
**Cytotoxicity LDH Assay Kit-WST**	DONJINDO	Cat# CK12
**RNeasy Mini Kit**	Qiagen	Cat# 74106

**PrimeScript™RT reagent Kit with gDNA Eraser**	TaKaRa	Cat# RR047A
**TB Green™ Premix Ex Taq™ II**	TaKaRa	Cat# RR820A
**DEPC H_2O**	Beyotime	Cat# R0022
**Cisplatin**	TOPSCIENCE	Cat# T1564
**Potassium Chloride**	Macklin	Cat# P816348
**Acetic acid**	Sigma-Aldrich	Cat# 695092
**Cell cycle Assay Kit(Red Fluorescence)**	Elabscience	E-CK-A351
**GlutaMAX**	Gibco	35050061
**Ham's F-12K (Kaighn's)**	Gibco	21127030
**Experimental models: cell lines**		
**CHO-K1**	National Collection of Authenticated Cell cultures	SCSP-507
**FaDu**	National Collection of Authenticated Cell cultures	TCHu132
**A549**	ATCC	CRM-CCL-185
**HEK-293T**	National Collection of Authenticated Cell cultures	SCSP-502
**Software and algorithms**		
**R**	https://www.r-project.org/ https://www.rstudio.com/	V4.0.2
**Python**	https://www.python.org/	V3.7.6
**UpSetR**	https://cran.rstudio.com/web/packages/UpSetR/	N/A
**ImageJ**	https://imagej.net/Welcome	V1.53
**Metascape**	https://metascN/Aape.org/	N/A
**Cytoscape**	https://cytoscape.org/	V3.9.1
**Gephi**	https://gephi.org/	V0.9.2
**GraphPad Prism v7**	https://www.graphpad.com/scientific-software/prism/	V7
**FlowJo**	https://www.flowjo.com/	V10.2
**Protein Atlas**	Uhlen et al., 2010	N/A
**BioGrid**	Stark et al., 2006	N/A
**InWeb_IM**	Li et al., 2017	N/A
**OmniPath**	Türei et al., 2016	N/A

**MCODE**	Bader and Hogue, 2003	N/A
**corrplot**	Wei et al., 2013	N/A
**pandas 1.0.1**	McKinney, 2011	N/A
**numpy 1.18.1**	Van Der Walt et al., 2011	N/A

RESOURCE AVAILABILITY

Lead contact

Further information and requests for resources and reagents should be directed to and will be fulfilled by the lead contact, Ning-Ning Liu (liuningning@shsmu.edu.cn).

Materials availability

This study did not generate new unique reagents.

Data and code availability

- **The code used in this paper can be freely accessed at https://github.com/MiGiNull/Ece1p-interactome.**
- **The Y2H screening results including interacting gene lists were collated in Supplementary Data 3 and 4.**
- **Any additional information required to reanalyze the data reported in this paper is available within the article and its supplemental information files, or is available from the lead contact upon request.**

Supplementary Table 2. Plasmids used in this study.

Plasmid Name	Bacterial Marker	Reference
**CCNH-3xFlag**	AmpR	This study
**Ece1-II-EGFP**	NeoR/KanR	This study
**Ece1-III-EGFP**	NeoR/KanR	This study
**Ece1-IV-EGFP**	NeoR/KanR	This study
**Ece1-V-EGFP**	NeoR/KanR	This study
**pBiFC-VC155**	AmpR	This study
**pBiFC-VN173**	AmpR	This study
**pBiFC-VC155-CCNH**	AmpR	This study
**pBiFC-VN173-Candidalysin**	AmpR	This study

Supplementary Table 3. The binding free energy of the docking pose decomposed to each residue. It could be observed in the following table that the Top 5 residues contributing the binding is Ile5, Ile3, Ile13, Pro14 and Gly4, which accords with the previous figure that these residues dominate the protein-peptide binding via hydrophobic interactions.

(VDW: Van der Waals interaction; ELE: electrostatic interaction; GB: polar solvation energy; SA: non-polar solvation energy).

RESIDUE_ID	VDW	ELE	GB	SA	TOTAL
Ile5	-3.37	-4.04	2.78	-0.53	-5.16
Ile3	-5.19	-1.39	2.99	-0.76	-4.35
Ile13	-4.26	1.53	-0.81	-0.61	-4.14
Pro14	-3.73	0.25	0.06	-0.58	-4
Gly4	-2.04	-7.68	6.56	-0.32	-3.48
Met7	-3.61	-1.04	2.4	-0.6	-2.83
Ile17	-1.79	1.25	-1.07	-0.44	-2.05
Ile2	-1.73	1.26	-1.11	-0.12	-1.7
Ile6	-1.1	-0.55	0.89	-0.14	-0.9
Ile9	-0.76	-0.77	0.9	-0.15	-0.78
Gly8	-0.89	-2.03	2.79	-0.23	-0.37
Gly11	-0.8	-1.82	2.39	-0.14	-0.36
Asn12	-1.75	0.35	1.29	-0.17	-0.28
Gln18	-0.51	-1.44	1.8	-0.13	-0.28
Met21	-0.22	0.59	-0.46	0	-0.09
Ile20	-0.14	0.87	-0.8	0	-0.07
Ile19	-0.05	0.8	-0.76	0	-0.01
Asn30	0	-2.27	2.26	0	-0.01
Ile23	-0.02	0.44	-0.42	0	0
Val24	-0.03	0.05	-0.03	0	0
Ala26	0	0.29	-0.28	0	0.01
Phe27	-0.01	0.09	-0.07	0	0.01
Gly29	0	-0.28	0.29	0	0.01
Ser22	-0.02	0.83	-0.77	0	0.04
Val16	-0.22	1.03	-0.74	0	0.07
Lys28	-0.01	4.82	-4.71	0	0.1
Lys25	-0.02	1.19	-1.06	0	0.11
Leu10	-0.21	0.28	0.04	0	0.12
Gln15	-0.6	1.11	-0.32	-0.06	0.14
Ser1	-2.73	1.23	11.12	-0.85	8.77

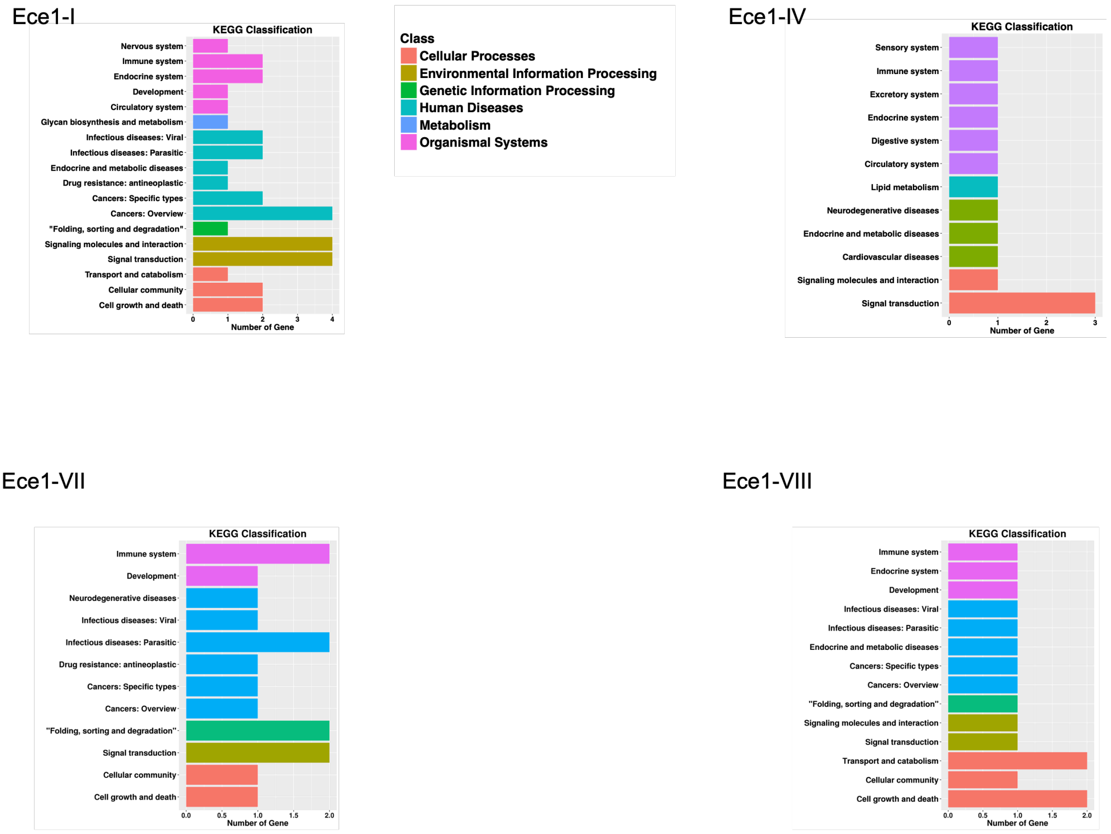

Supplementary Fig. 1. KEGG enrichment analysis of RNA-seq data from Ece1 peptides (Ece1-I, IV, VII, VIII) infected cells.

Histograms of KEGG enrichment analysis associated with Ece1-I, IV, VII and VIII. The top significantly enriched features and the corresponding gene numbers are shown.

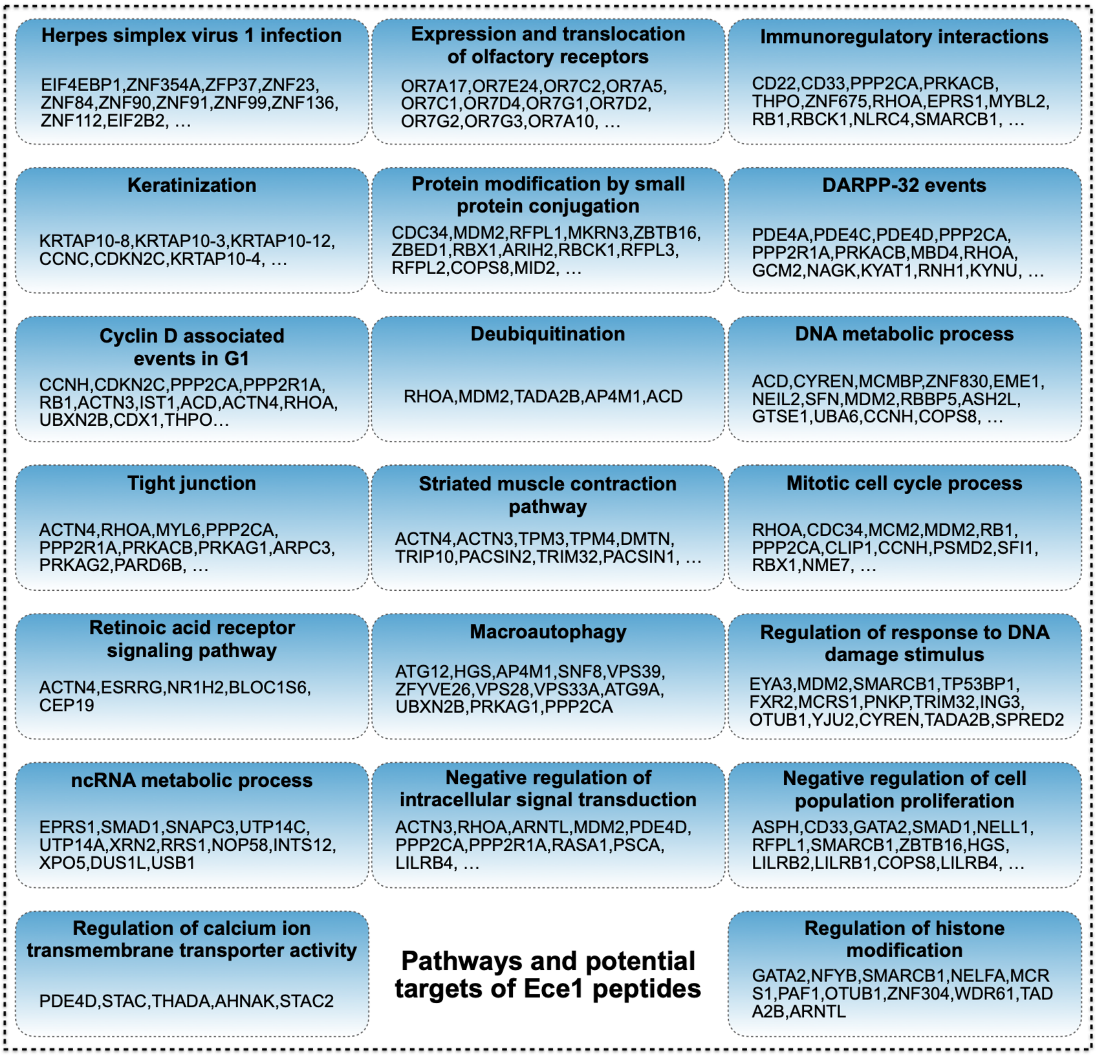

Supplementary Fig. 2. The twenty representative pathways and corresponding potential targets of Ece1 peptides, related to Fig. 3.

The potential targets of Ece1 peptides could be enriched in various biological functions and pathways.

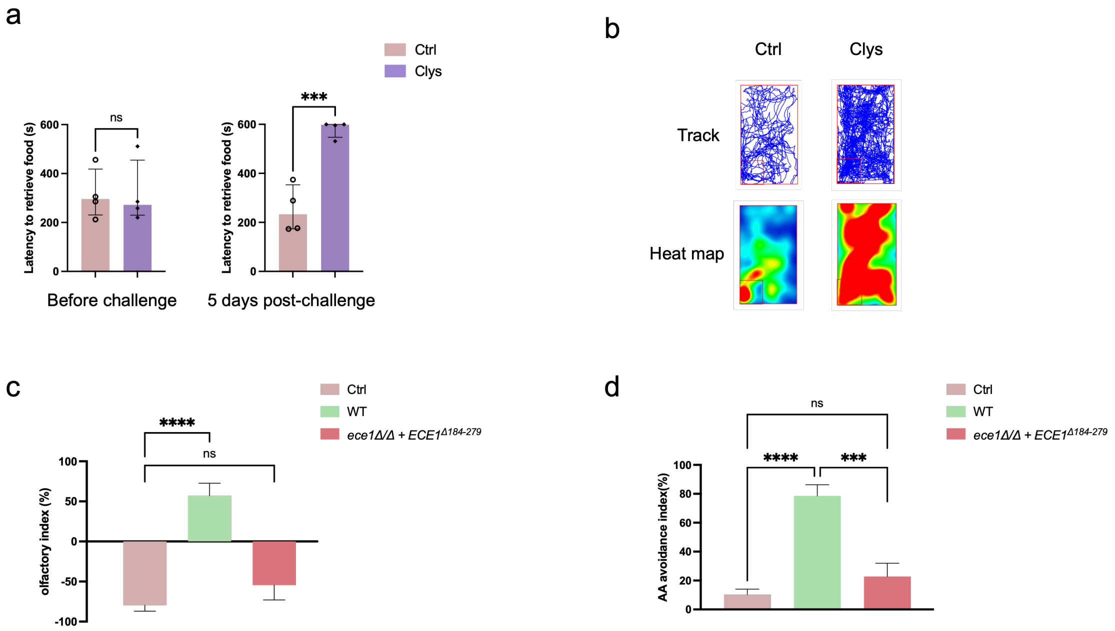

Supplementary Fig 3. Candidalysin impairs the olfactory function in animals.

(a) Latency to retrieve food of mice in the food-burial search test before and after treated with candidalysin or PBS (Ctrl). The mice infected with candidalysin spent a significantly longer time before retrieving the buried food. $N = 4$ mice per group.

(b) Track plot and heat map of mice in the process of locating and retrieving buried food. The size of the cage was 16×30 cm.

(c & d) Quantification of male olfactory responses toward acetic acid (AA) diluted in distilled water (10% v/v) in Y-maze assay. (c) Olfactory index was calculated using the following formula: olfactory index = (number in the odor tube - number in the solvent tube)/total number of loaded flies. (d) AA avoidance index = number in the odor tube/ total number of loaded flies. $N = 4$, representing a total of 81-143 flies.

Data are presented as mean ± SEM. ns, not significant, $*P < 0.05$, $**P < 0.01$, $***P < 0.001$, $****P < 0.0001$, as determined by unpaired two-tailed Student's t-test (a), one-way ANOVA test with Tukey correction (c, d).

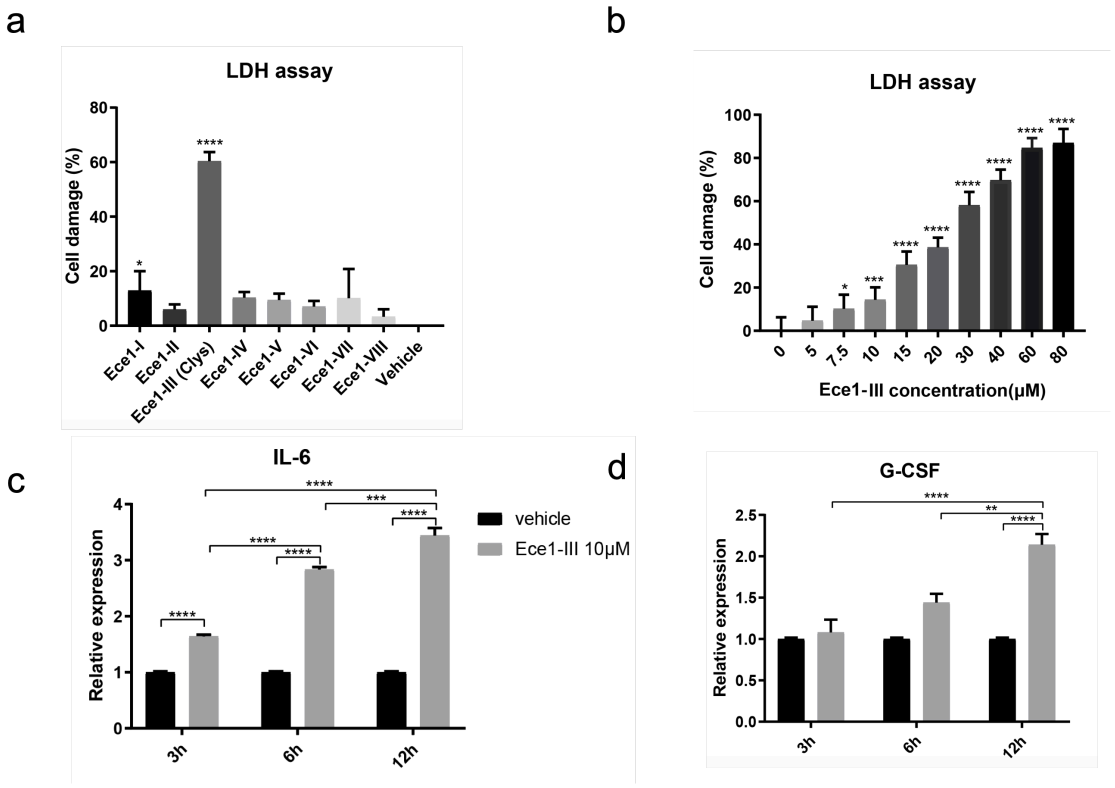

Supplementary Fig. 4. Candidalysin induces cell damage and production of cytokines, related to Fig. 4.

(a) LDH release after incubation of FaDu cells with Ece1 peptides.

(b) LDH release of FaDu cells after treatment with serial dilution concentrations of Ece1-III (0-80 μM).

(c) IL-6 expression after incubation with 10 μM Ece1-III within 12h.

(d) G-CSF expression after incubation with 10 μM Ece1-III within 12h.

Data are presented as mean ± SEM. ns, not significant, **P* < 0.05, ***P* < 0.01, ****P* < 0.001, *****P* < 0.0001 as determined by one-way ANOVA test with Tukey correction (a, b), unpaired two-tailed Student's t-test (c, d).

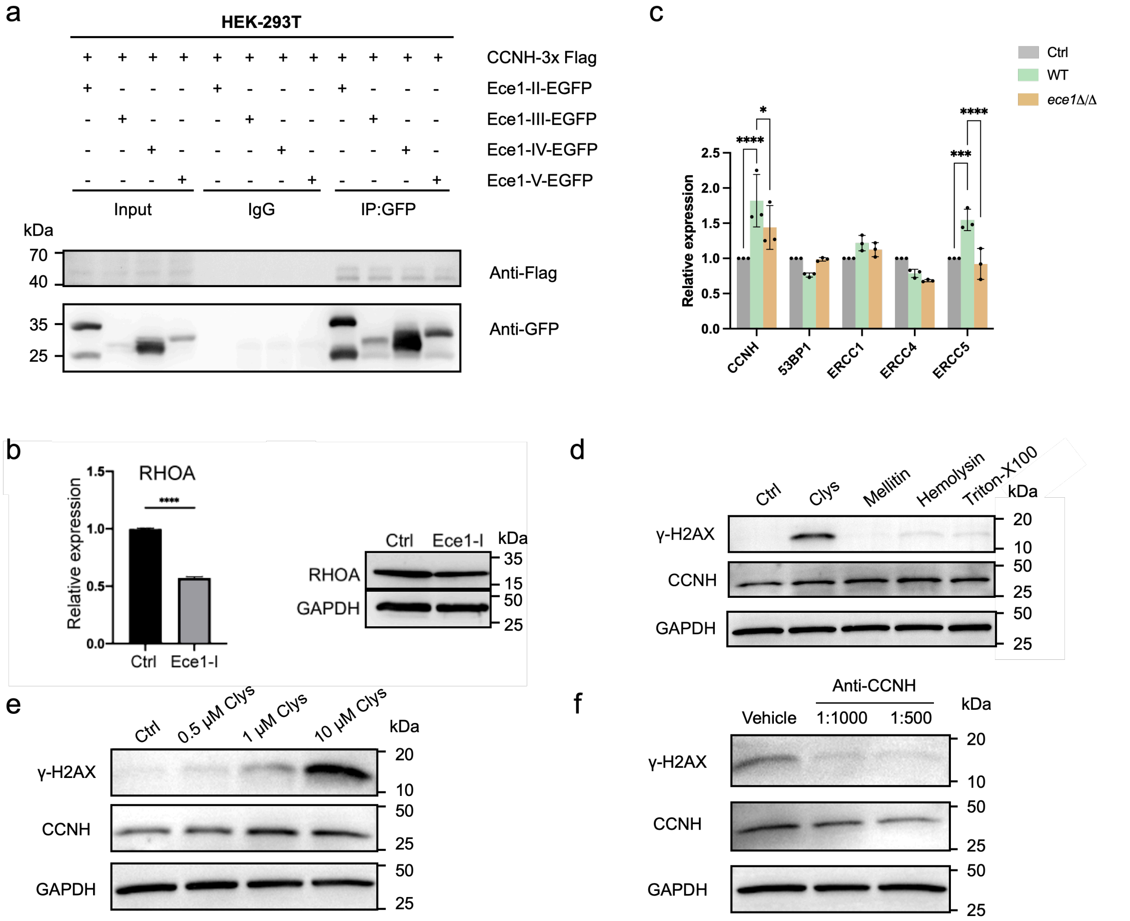

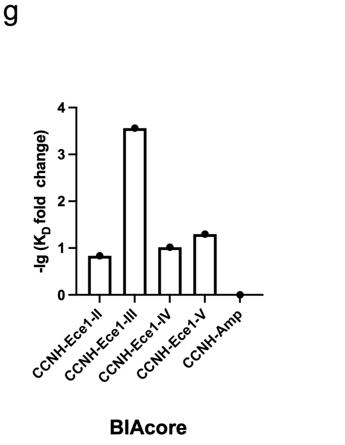

h

Ligand	Receptor	Dynamic Parameters Ka (/Ms)	Kd (/s)	KD (M)	Chi^2
Ece1-II		3.16E-06	4.15E-03	9.13E-06	4.34E-02
Ece1-III		9.92E+04	1.71E-03	1.72E-08	3.58E-03
Ece1-IV	CCNH	4.98E+01	2.97E-04	5.98E-06	8.41E-02
Ece1-V		2.28E+02	7.19E-04	3.16E-06	3.41E-01
Amp		3.04E+03	1.90E-01	6.26E-05	3.79E+00

Supplementary Fig. 5. Validation of interaction between Ece1 peptides and CCNH. Candidalysin induces DNA double strand breaks and suppresses DNA damage repair by binding directly to CCNH, related to Fig. 4.

(a) Co-immunoprecipitation assay demonstrated the interaction between intracellular CCNH and Ece1 peptides (Ece1-II to V).

(b) Expression levels (left, mRNA level; right, protein level) of RhoA in FaDu cells stimulated by 15 μM Ece1-I peptide for 4h.

(c) Expression levels of DNA damage repair associated genes (53BP1, ERCC1, ERCC2, ERCC4) were measured via qRT-PCR after co-culturing cells with the *C. albicans* WT and *ece1Δ/Δ*. Data are presented as mean ± SEM. ns, not significant, *, $P = 0.0109$; ***, $P = 0.0003$; ****, $P < 0.0001$, as determined by two-way ANOVA test with Tukey correction.

(d) Expression levels of CCNH and γ-H2AX in FaDu cells following treatments of Clys (candidalysin), mellitin, hemolysin and triton-X100.

(e) Expression levels of CCNH and γ-H2AX in FaDu cells following treatments of different concentrations of Clys (0.5 μM, 1 μM, 10 μM).

(f) Blocking of CCNH led to a suppressed expression of γ-H2AX within the FaDu cells.

(g) The binding of Ece1-II to V peptides and Amp to CCNH through surface plasmon resonance assays (via BIAcore). Amp, human cathelicidin (FKRIVQRIKDFLRNLVPRTES). K_D fold change = K_D(CCNH-peptide)/ K_D(CCNH-Amp).

(h) Data of BIAcore analysis, related to Supplementary Fig. 4g. K_a, the binding rate constant; K_d, dissociation rate constant; K_D, equilibrium dissociation constant; Chi^2, square of relative response (RU).

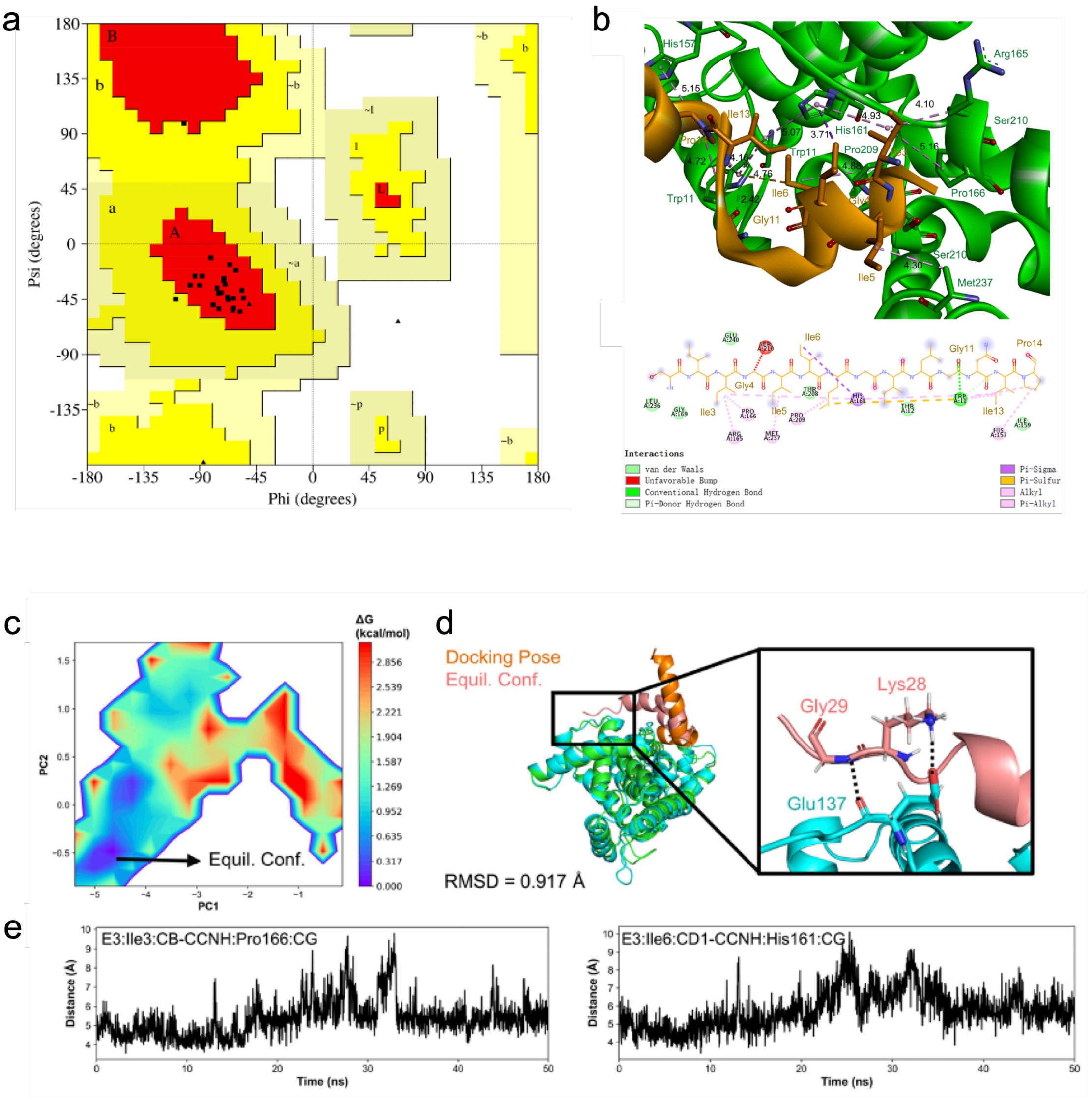

Supplementary Fig 6. Molecular docking analysis of candidalysin and CCNH, related to Fig. 4.

(a) The Ramachandran plot plotted using SAVES v6.0. 91.3% of the residues locate in the most favored regions, above the recommended 90%. The plot suggests the validity of backbone torsions.

(b) CCNH-candidalysin binding interface. Zoomed 3D (upper panel) and 2D (lower panel) interaction plots. The binding is mostly triggered by hydrophobic interaction, but not a moderate H-bond between Trp11 on CCNH and Gly11 on candidalysin. The relative residues and distance of interactions are labeled.

(c) The trajectory was processed with principal component analysis (PCA). The equilibrated conformation (Equil. Conf.) was extracted as the conformation around (-4.6, -0.5).

(d) The equilibrated conformation of candidalysin is almost the same of the docking pose.

(e) The distances representing hydrophobic interactions in the docking pose was monitored. These distances are mostly stably maintained around those in the docking pose, suggesting the stability of the peptide-protein interactions.

Supplementary Texts

Supplementary information about verification of modeled structures and analysis of CCNH-candidalysin binding.

Molecular docking of candidalysin to CCNH was performed with the HPepDock server[1] (http://huanglab.phys.hust.edu.cn/hpepdock/). The structure of CCNH was fetched from Protein Data Bank[2] (PDB ID: 1KXU[3]), while the docking pose of candidalysin was sampled by the server based on the input sequence. Note that the only first 30 residues are selected for modeling due to restrictions by the webserver. Therefore, it would be worthy to evaluate the structural validity of the docking pose of candidalysin.

(1) Ramachandran plot

The Ramachandran plot[4] (Supplementary Fig. 5a) was plotted using SAVES v6.0 (https://saves.mbi.ucla.edu/). It could be observed that 91.3% of the residues locate in the most favored regions, above the recommended 90%. No residue locates in disallowed regions. The plot suggests the validity of backbone torsions.

(2) ERRAT Score

The ERRAT score[5] is calculated using the SAVES v6.0 webserver. The score has a value of 68.1818, suggesting a moderate good standard of structure from the perspective of crystallography.

Based on previous analyses, it could be inferred that sampled docking pose by HPepDock server is reasonable in structure.

Analysis of CCNH-candidalysin Binding

(1) CCNH-candidalysin binding interface

To take a deep insight into the CCNH-candidalysin binding interactions, we first analyzed the interaction surface. The analysis was done using Discovery Studio 2016 and 3D (Supplementary Fig. 5b, upper) as well as 2D (Supplementary Fig. 5b, lower) interaction plots are shown below. It could be observed that the binding is mostly triggered by hydrophobic interaction, instead a moderate H-bond between Trp11 on CCNH and Gly11 on candidalysin. Relative residues and distance of interactions are labeled in the upper panel. Types of interactions are listed in the lower panel.

(2) MM/GBSA study for energetical analysis

Then, we performed MM/GBSA study for energetical analysis of peptide binding using the HawkDock server[6] (http://cadd.zju.edu.cn/hawkdock/). The binding free energy was calculated for the docking pose and was further decomposed to each residue. It could be observed in the Supplementary Table 4 that the top 5 residues contributing the binding is Ile5, Ile3, Ile13, Pro14 and Gly4, which accords with Supplementary Fig. 5b that these residues dominate the protein-peptide binding via hydrophobic interactions.

(3) Short time Molecular Dynamic simulation

Finally, we performed molecular dynamic simulation to evaluate the stability of the docking pose. The complex was solvated with a TIP3P box and neutralized with *tleap* in AMBER18[7] package. The system was minimized and then heated to 300K in NVT ensemble within 100 ps. After that, the system was equilibrated in turn in NVT and NPT ensemble, each for 100 ps. Finally, the system was simulated for 50 ns using NPT ensemble. The minimization and simulation were performed with the GPU-accelerated form of *pmemd* in AMBER18[7] package using the ff14SB[8] forcefield.

The trajectory was processed with principal component analysis (PCA). The equilibrated conformation (Equil. Conf.) was extracted as the conformation around (-4.6, -0.5) in Supplementary Fig. 5c. It could be observed in Supplementary Fig. 5d that the equilibrated conformation of the peptide is almost the same as that of the docking

pose. Instead, the C-terminal of the peptide forms two additional H-bonds with CCNH, causing the previous erect N-terminal fall down to the surface of CCNH. This is rational because in molecular docking, solvation effects could not be considered. Besides, we have monitored the distances representing hydrophobic interactions in the docking pose. It could be seen in Supplementary Fig. 5e that these distances are mostly and stably maintained around those in the docking pose, suggesting the stability of the peptide-protein interactions.

Uncropped scans of source western blot gels related to supplementary figures

Supplementary Fig. 5b

Ctrl Ece1-I

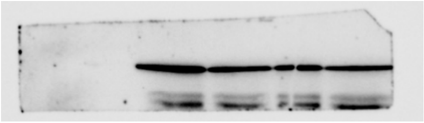

RHOA

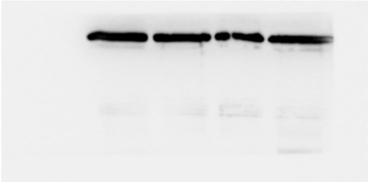

GAPDH

Supplementary Fig. 5d

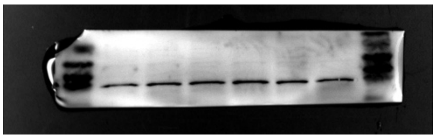

CCNH

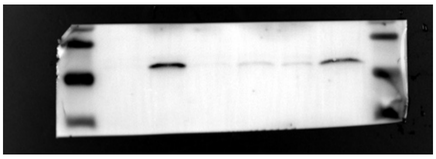

γ-H2AX

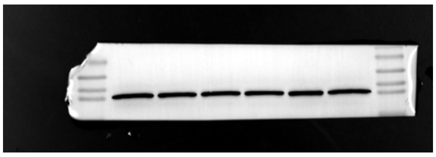

GAPDH

Supplementary Fig. 5e

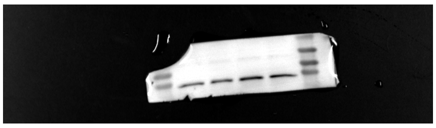

CCNH

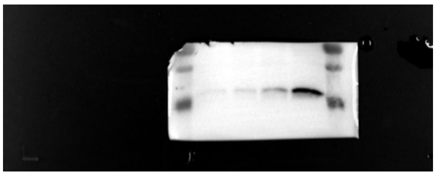

γ-H2AX

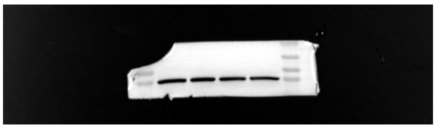

GAPDH

Supplementary Fig. 5f

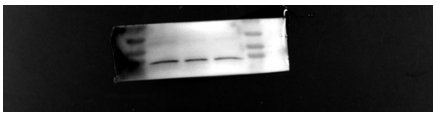

CCNH

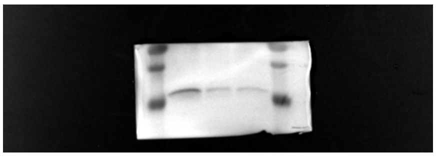

γ-H2AX

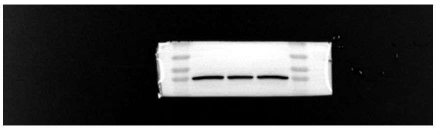

GAPDH

Supplementary References

1 Zhou, P., Jin, B. W., Li, H. & Huang, S. Y. HPEPDOCK: a web server for blind peptide-protein docking based on a hierarchical algorithm. *Nucleic Acids Res* **46**, W443-W450 (2018). https://doi.org:10.1093/nar/gky357

2 Burley, S. K. *et al.* RCSB Protein Data Bank (RCSB.org): delivery of experimentally-determined PDB structures alongside one million computed structure models of proteins from artificial intelligence/machine learning. *Nucleic Acids Res* **51**, D488-D508 (2023). https://doi.org:10.1093/nar/gkac1077

3 Kim, K. K., Chamberlin, H. M., Morgan, D. O. & Kim, S. H. Three-dimensional structure of human cyclin H, a positive regulator of the CDK-activating kinase. *Nat Struct Biol* **3**, 849-855 (1996). https://doi.org:DOI 10.1038/nsb1096-849

4 Laskowski, R. A., Macarthur, M. W., Moss, D. S. & Thornton, J. M. Procheck - a Program to Check the Stereochemical Quality of Protein Structures. *J Appl Crystallogr* **26**, 283-291 (1993). https://doi.org:Doi 10.1107/S0021889892009944

5 Colovos, C. & Yeates, T. O. Verification of Protein Structures - Patterns of Nonbonded Atomic Interactions. *Protein Sci* **2**, 1511-1519 (1993). https://doi.org:DOI 10.1002/pro.5560020916

6 Weng, G. Q. *et al.* HawkDock: a web server to predict and analyze the protein-protein complex based on computational docking and MM/GBSA. *Nucleic Acids Res* **47**, W322-W330 (2019). https://doi.org:10.1093/nar/gkz397

7 Case, D. A. *et al.* AMBER 2018, University of California, San Francisco. (2018).

8 Maier, J. A. *et al.* ff14SB: Improving the Accuracy of Protein Side Chain and Backbone Parameters from ff99SB. *J Chem Theory Comput* **11**, 3696-3713 (2015). https://doi.org:10.1021/acs.jctc.5b00255
