# Supplementary material for: Global fungal-host interactome mapping identifies host targets of candidalysin
Source: Nat Commun. 2024 Feb 27;15:1757. doi: 10.1038/s41467-024-46141-x (PMC10899660; doi:10.1038/s41467-024-46141-x)
Supplement: Supplementary file 3 — Description of Additional Supplementary Files [file 41467_2024_46141_MOESM3_ESM.pdf]

Description of Additional Supplementary Files

File Name: Supplementary Data 1
Description: Lists of differentially expressed genes associated with Ece1-I, Ece1-IV, Ece1-VII and Ece1-VIII, related to Supplementary Fig. 1.

File Name: Supplementary Data 2
Description: KEGG class enrichment results associated with Ece1-I, Ece1-IV, Ece1-VII and Ece1-VIII, related to Supplementary Fig. 1.

File Name: Supplementary Data 3
Description: List of human interactors for each Ece1 peptide, related to Fig. 2.

File Name: Supplementary Data 4
Description: Bioinformatical results via Metascape, including annotation and enrichment, related to Fig. 3.
