# Supplementary material for: Global fungal-host interactome mapping identifies host targets of candidalysin
Source: Nat Commun. 2024 Feb 27;15:1757. doi: 10.1038/s41467-024-46141-x (PMC10899660; doi:10.1038/s41467-024-46141-x)
Supplement: Supplementary file 8 — Reporting Summary [file 41467_2024_46141_MOESM8_ESM.pdf]

nature portfolio

Corresponding author(s): Ningning Liu, Changbin Chen, Hui Wang

Last updated by author(s): Feb 4, 2024

Reporting Summary

Nature Portfolio wishes to improve the reproducibility of the work that we publish. This form provides structure for consistency and transparency in reporting. For further information on Nature Portfolio policies, see our Editorial Policies and the Editorial Policy Checklist.

Statistics

For all statistical analyses, confirm that the following items are present in the figure legend, table legend, main text, or Methods section.

n/a	Confirmed	
☐	☒	The exact sample size (*n*) for each experimental group/condition, given as a discrete number and unit of measurement
☐	☒	A statement on whether measurements were taken from distinct samples or whether the same sample was measured repeatedly
☐	☒	The statistical test(s) used AND whether they are one- or two-sided<br>*Only common tests should be described solely by name; describe more complex techniques in the Methods section.*
☐	☒	A description of all covariates tested
☐	☒	A description of any assumptions or corrections, such as tests of normality and adjustment for multiple comparisons
☐	☒	A full description of the statistical parameters including central tendency (e.g. means) or other basic estimates (e.g. regression coefficient) AND variation (e.g. standard deviation) or associated estimates of uncertainty (e.g. confidence intervals)
☐	☒	For null hypothesis testing, the test statistic (e.g. *F*, *t*, *r*) with confidence intervals, effect sizes, degrees of freedom and *P* value noted<br>*Give P values as exact values whenever suitable.*
☒	☐	For Bayesian analysis, information on the choice of priors and Markov chain Monte Carlo settings
☒	☐	For hierarchical and complex designs, identification of the appropriate level for tests and full reporting of outcomes
☐	☒	Estimates of effect sizes (e.g. Cohen's *d*, Pearson's *r*), indicating how they were calculated

Our web collection on statistics for biologists contains articles on many of the points above.

Software and code

Policy information about availability of computer code

Data collection	The global interactome data are from a high-throughput enhanced yeast two-hybrid (HT-eY2H) screening between Ecel peptides and the human ORFeome.
Data analysis	ImageJ (v 1.53), R (v 4.0.2), Python (v 3.7.6), UpSetR, corrplot, pandas (v 1.0.1), numpy (v 1.18.1), Metascape, Cytoscape (v 3.9.1.), Gephi (v 0.9.2), GraphPad Prism (v 7.0), FlowJo (v 10.2).

For manuscripts utilizing custom algorithms or software that are central to the research but not yet described in published literature, software must be made available to editors and reviewers. We strongly encourage code deposition in a community repository (e.g. GitHub). See the Nature Portfolio guidelines for submitting code & software for further information.

Data

Policy information about availability of data

All manuscripts must include a data availability statement. This statement should provide the following information, where applicable:

- Accession codes, unique identifiers, or web links for publicly available datasets
- A description of any restrictions on data availability
- For clinical datasets or third party data, please ensure that the statement adheres to our policy

All datasets and raw data generated and/or analyzed during the current study are available from the corresponding author upon reasonable request. All data needed to evaluate the conclusions in the paper, including data associated with main figures and supplementary figures, are available within the article or in the

Supplementary Information, Source Data and Extended Data. Any additional information required to reanalyze the data reported in this paper is available from the lead contact upon request. The raw data used for mRNA-seq analysis is available at NCBI under accession number BioProjectID: PRJNA1051990 and will be made publicly available upon publication. The code used in this paper can be freely accessed at https://github.com/MiGiNull/Ece1p-interactome.

Research involving human participants, their data, or biological material

Policy information about studies with human participants or human data. See also policy information about sex, gender (identity/presentation), and sexual orientation and race, ethnicity and racism.

Reporting on sex and gender	NA
Reporting on race, ethnicity, or other socially relevant groupings	NA
Population characteristics	NA
Recruitment	NA
Ethics oversight	NA

Note that full information on the approval of the study protocol must also be provided in the manuscript.

Field-specific reporting

Please select the one below that is the best fit for your research. If you are not sure, read the appropriate sections before making your selection.

☒ Life sciences ☐ Behavioural & social sciences ☐ Ecological, evolutionary & environmental sciences

For a reference copy of the document with all sections, see nature.com/documents/nr-reporting-summary-flat.pdf

Life sciences study design

All studies must disclose on these points even when the disclosure is negative.

Sample size	No sample-size calculations were performed. As indicated in the text, for all cells, each experimental group includes more than 5*10^5 cells, for mice, each experimental group includes more than 6 animals, and for flies, each experimental group includes more than 80 animals. For flow cytometry, we analyzed 10000 cells / sample. The cell suspension is introduced into the flow cytometer, where it is typically constricted into a very narrow stream within the instrument, allowing cells to pass through the detection area one by one. The flow cytometer analyzes individual cells using lasers and detectors, enabling rapid and accurate cell counting and sorting.
Data exclusions	No data was excluded from the analyses.
Replication	Each experiments were replicated more than 3 times and all attempts at replicating assay were successful.
Randomization	Animals were randomized into cages by vivarium staff and randomly assigned to each group.
Blinding	Since no population-based experiments were involved in this study, binding is not applicable for this study.

Reporting for specific materials, systems and methods

We require information from authors about some types of materials, experimental systems and methods used in many studies. Here, indicate whether each material, system or method listed is relevant to your study. If you are not sure if a list item applies to your research, read the appropriate section before selecting a response.

Materials & experimental systems

n/a	Involved in the study	
☐	☒	Antibodies
☐	☒	Eukaryotic cell lines
☒	☐	Palaeontology and archaeology
☐	☒	Animals and other organisms
☒	☐	Clinical data
☒	☐	Dual use research of concern
☒	☐	Plants

Methods

n/a	Involved in the study	
☒	☐	ChIP-seq
☐	☒	Flow cytometry
☒	☐	MRI-based neuroimaging

Antibodies

Antibodies used

CCNH antibody (CST,Rabbit Source, Cat#2927, 1:1000 for WB, 1:1000 and 1:500 for blockade experiment, 1:500 for Immunofluoresence assay)
gama-H2AX antibody (CST, Rabbit Source, Cat#97185, 1:1000 for WB)
Anti-gamma H2A.X (phospho S139) antibody (Abcam, Mouse Source, Cat#ab303656, 1:500 for Immunofluoresence assay)
GAPDH (D16H11) XP® Rabbit mAb (CST, Rabbit Source, Cat#5174S, 1:1000 for WB)
Anti-Cyclin H/p34 antibody (Abcam, Rabbit Source, Cat#EPR3929, 1:100 for histopathology)
Anti-CDK2 (phospho T160) + CDK1 (phospho T161) antibody (Abcam, Rabbit Source, Cat#EPR17621, 1:1000 for WB)
Anti-rabbit IgG, HRP-linked Antibody (CST, Goat Source, Cat#7074, 1:5000 for WB)
Goat Anti-Mouse IgG H&L (Alexa Fluor® 647) (Abcam, Goat Source, Cat#ab150115,1:500 for Immunofluoresence assay)
Goat Anti-Rabbit IgG H&L (Alexa Fluor® 488) (Abcam, Goat Source, Cat#ab150077, 1:500 for Immunofluoresence assay)
Mouse IgG (Magnetic Bead Conjugate) (CST, Mouse Source, Cat#5873, 1:10 for Immunoprecipitation)
DYKDDDDK Tag (D6W5B) Rabbit mAb (CST, Rabbit Source, Cat#14793, 1:1000 for WB)
GFP (5G4) Mouse mAb (Magnetic Bead Conjugate) (CST, Mouse Source, Cat#67090, 1:10 for Immunoprecipitation)
GFP (4B10) Mouse mAb (CST, Mouse Source, Cat #2955, 1:1000 for WB)
Anti-mouse IgG,HRP-linked antibody (CST, Goat Source, Cat#7076, 1:5000 for WB)

Validation

All antibodies are used in this manuscript are well-established, commercially available antibodies. Validations for each of these commercial antibodies are provided on the manufacturers' websites and include specificity and quality control testing via flow cytometry and testing for contaminants including endotoxin. The specific antibody information are as follows.
CCNH antibody (CST,Cyclin H Antibody detects endogenous levels of cyclin H. It does not cross-react with other family members at physiological levels.https://www.cellsignal.cn/product/productDetail.jsp?productId=2927)
gama-H2AX antibody (CST, Phospho-Histone H2A.X (Ser139) (20E3) Rabbit mAb detects endogenous levels of H2A.X only when phosphorylated at Ser139. https://www.cellsignal.cn/product/productDetail.jsp?productId=9718)
Anti-gamma H2A.X (phospho S139) antibody (Abcam, Produced recombinantly (animal-free) for high batch-to-batch consistency and long term security of supply, https://www.abcam.cn/products/primary-antibodies/gamma-h2ax-phospho-s139-antibody-n1-431-ab303656.html)
GAPDH (D16H11) XP® Rabbit mAb (CST, GAPDH (D16H11) XP® Rabbit mAb detects endogenous levels of total GAPDH protein. https://www.cellsignal.cn/product/productDetail.jsp?productId=5174)
Anti-Cyclin H/p34 antibody (Abcam, The Abpromise guarantee)
Anti-CDK2 (phospho T160) + CDK1 (phospho T161) antibody (Abcam, Produced recombinantly (animal-free) for high batch-to-batch consistency and long term security of supply, https://www.abcam.cn/products/primary-antibodies/cdk2-phospho-t160--cdk1-phospho-t161-antibody-epr17621-ab183554.html)
Anti-rabbit IgG, HRP-linked Antibody (CST, This product is thoroughly validated with CST primary antibodies and will work optimally with the CST western immunoblotting protocol, ensuring accurate and reproducible results.https://www.cellsignal.cn/product/productDetail.jsp?productId=7074)
Goat Anti-Mouse IgG H&L (Alexa Fluor® 647) (Abcam, The Abpromise guarantee, https://www.abcam.cn/products/secondaryantibodies/
goat-mouse-igg-hl-alexa-fluor-647-ab150115.html)
Goat Anti-Rabbit IgG H&L (Alexa Fluor® 488) (Abcam, The Abpromise guarantee, https://www.abcam.cn/products/secondaryantibodies/
goat-rabbit-igg-hl-alexa-fluor-488-ab150077.html)
Mouse IgG (Magnetic Bead Conjugate) (CST,This Cell Signaling Technology normal mouse IgG (whole molecule) containing predominantly IgG1 and IgG2a isotypes and small amounts of IgG2b and IgG3 is immobilized by the covalent reaction of hydrazinonicotinamide-modifed antibody with formylbenzamide-modified magnetic beads. Mouse IgG (Magnetic Bead Conjugate) is useful to determine non-specific immunoprecipitation complexes. https://www.cellsignal.cn/products/wb-ip-reagents/mouse-iggmagnetic-
bead-conjugate/5873)
DYKDDDDK Tag (D6W5B) Rabbit mAb (CST, DYKDDDDK Tag (D6W5B) Rabbit mAb detects exogenously expressed DYKDDDDK proteins in cells. The antibody recognizes the DYKDDDDK peptide, which is the same epitope recognized by Sigma-Aldrich Anti-FLAG M2 antibody, fused to either the amino-terminus or carboxy-terminus of the target protein. https://www.cellsignal.com/product/productDetail.jsp?productId=14793&country=AS)
GFP (5G4) Mouse mAb (Magnetic Bead Conjugate) (CST,This Cell Signaling Technology antibody is immobilized by the covalent reaction of formylbenzamide-modified antibody with hydrazide-activated magnetic bead. GFP (5G4) Mouse mAb (Magnetic Bead Conjugate) is useful for immunoprecipitation assays of GFP-tagged recombinant proteins. This antibody conjugate is expected to exhibit the same species cross-reactivity as the unconjugated GFP (5G4) Mouse mAb. https://www.cellsignal.cn/products/antibodyconjugates/
gfp-5g4-mouse-mab-magnetic-bead-conjugate/67090)
GFP (4B10) Mouse mAb (CST, GFP (4B10) Mouse mAb detects GFP, YFP, and CFP-tagged proteins exogenously expressed in cells. This antibody does not detect RFP-tagged proteins. Please note that the GFP, YFP, and CFP tags add approximately 27 kDa to the molecular weight of the fusion protein.https://www.cellsignal.cn/products/primary-antibodies/gfp-4b10-mouse-mab/2955)
Anti-mouse IgG,HRP-linked antibody (CST, Affinity purified horse anti-mouse IgG (heavy and light chain) antibody is conjugated to horseradish peroxidase(HRP) for chemiluminescent detection. This product is thoroughly validated with CST primary antibodies and will work optimally with the CST western immunoblotting protocol, ensuring accurate and reproducible results. https://www.cellsignal.com/product/productDetail.jsp?productId=7076)

Eukaryotic cell lines

Policy information about cell lines and Sex and Gender in Research

Cell line source(s)

FaDu cell line, CHO-K1 cell line, A549 cell line and HEK293T cell line.

Authentication	The cell lines FaDu, CHO-K1 and HEK293T were obtained from Stem Cell Bank, Chinese Academy of Sciences. The cell lines A549 were obtained from the ATCC.
Mycoplasma contamination	All cell lines tested negative for mycoplasma contamination.
Commonly misidentified lines (See ICLAC register)	No commonly misidentified cell lines were used and all cell lines were tested by STR profiling.

Animals and other research organisms

Policy information about studies involving animals; ARRIVE guidelines recommended for reporting animal research, and Sex and Gender in Research

Laboratory animals	Mice, BALB/c, female, 8weeks. Mice had a 12h light cycle per day with housing conditions at maintained at 68–74°F and 30–70% humidity.<br>Flies, w1118, female, 3 to 5 days.
Wild animals	The study did not involve wild animals.
Reporting on sex	The study did not involve sex-based analysis.
Field-collected samples	The study did not involve samples collected from the field.
Ethics oversight	All the procedures were conducted in compliance with a protocol approved by the Institutional Animal Care and Use Committee at Institute Pasteur of Shanghai, CAS.

Note that full information on the approval of the study protocol must also be provided in the manuscript.

Plants

Seed stocks	NA
Novel plant genotypes	NA
Authentication	NA

Flow Cytometry

Plots

Confirm that:

☒ The axis labels state the marker and fluorochrome used (e.g. CD4-FITC).

☒ The axis scales are clearly visible. Include numbers along axes only for bottom left plot of group (a 'group' is an analysis of identical markers).

☒ All plots are contour plots with outliers or pseudocolor plots.

☒ A numerical value for number of cells or percentage (with statistics) is provided.

Methodology

Sample preparation	The CHO-K1 cells were collected and fixed overnight in 70% ethanol at 20 °C, washed and resuspended in 100 g/mL RNase A Reagent for 30 min at 37°C, then treated with 15 g/mL propidium iodide at 4°C for 30 min in dark condition. After staining, cell-cycle analyses were carried out by flow cytometry.
Instrument	BECKMAN COULTER, Inc. CytoFLEX LX, Model NO. B90883.
Software	CytExpert were used to collect data and Flowjo were used to analyze data.
Cell population abundance	After collecting more than 10000 single cells per group, we divided the single cells into G1 phase, G2 phase, and S phase according to the fluorescence intensity of the FL10 channel.
Gating strategy	In our gating strategy for the starting cell population, we set preliminary gates based on forward scatter (FSC) and side scatter (SSC) properties to identify cell population. We first used an unstained control sample to set FSC and SSC to clearly

delineate cell populations and exclude dead cells and debris. To define the boundaries between "positive" and "negative" staining cell populations, we used unstained samples and isotype controls from the same donor as negative controls. Compensation controls were included in each experiment to correct for spectral overlap of the fluorochromes. The delineation of positive and negative cell populations was determined based on the baseline fluorescence intensity levels of these negative control samples.

☒ Tick this box to confirm that a figure exemplifying the gating strategy is provided in the Supplementary Information.
