# Supplementary material for: Global fungal-host interactome mapping identifies host targets of candidalysin
Source: Nat Commun. 2024 Feb 27;15:1757. doi: 10.1038/s41467-024-46141-x (PMC10899660; doi:10.1038/s41467-024-46141-x)

**Source data of Fig. 1. Plate dotting images of the 2^nd^ HT-eY2H screening of the 8 Ece1 derived peptides on two different types of selective media.**

Columns 1-6 of Controls: 1 expresses DB and AD plasmids without any fusion; 2 expresses DB-pRB and AD-E2F1 fusion proteins, forming an interaction, and is CHX-sensitive; 3, 4, and 5 express DB-Fos and AD-Jun, DB-GAL4 and AD, and DB-DP and AD-E2F1, respectively, all of which exhibit positive interactions but are CHX-resistant; 6 expresses DB-DP and AD-E2F1, and is CHX-sensitive. The annotation of 1-12 and A-H facilitates our localization of each protein. The colonies were color-categorized according to growth intensity as very strong (red), strong (orange), medium (yellow), weak (cyan), and very weak (blue). Auto-interactive colonies were marked with black squares. Ctrl, Control.


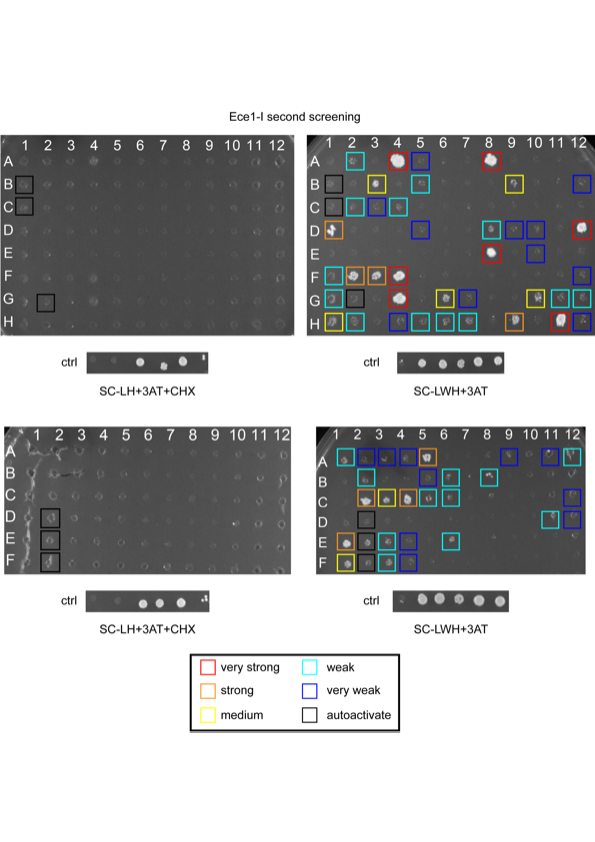

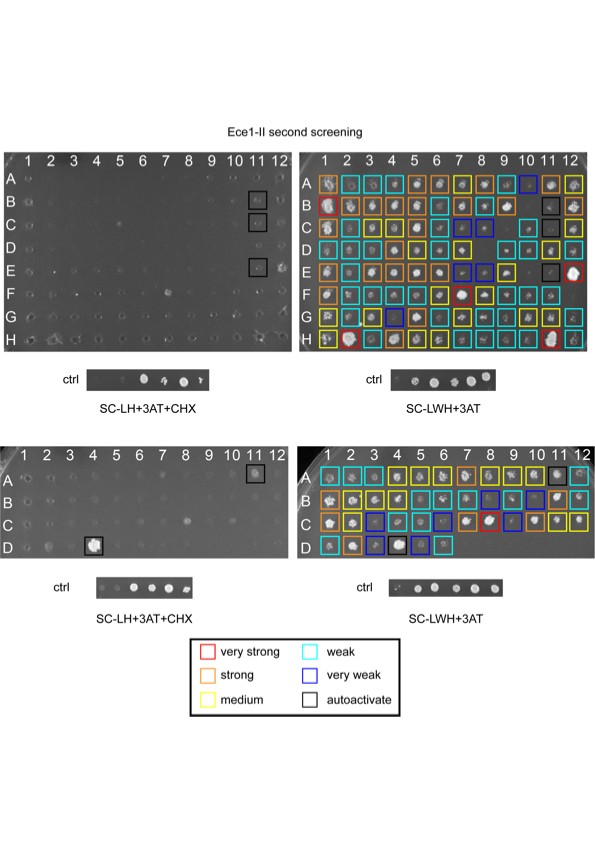

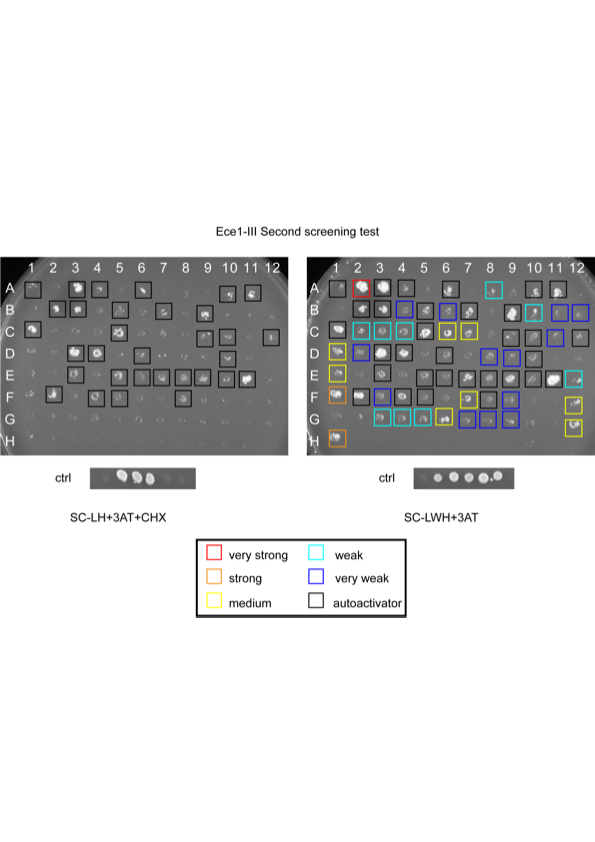

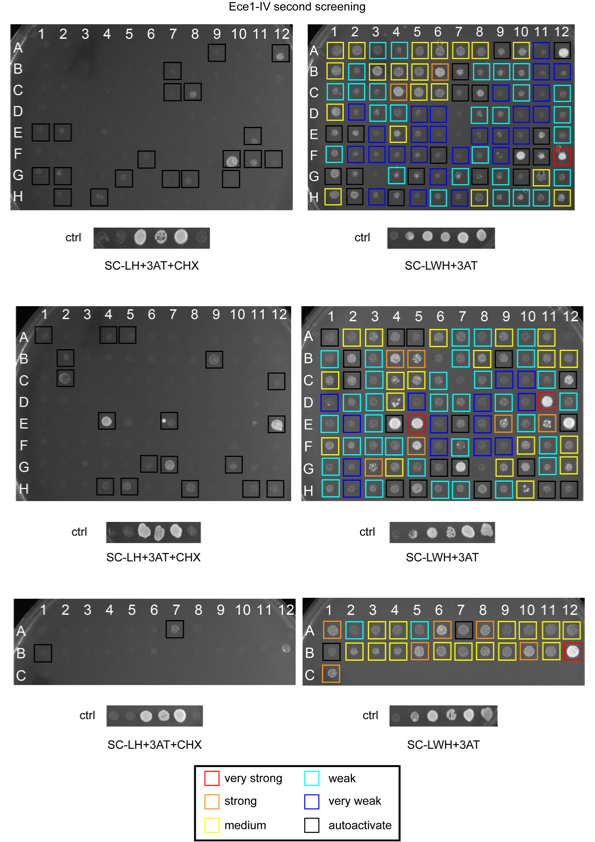

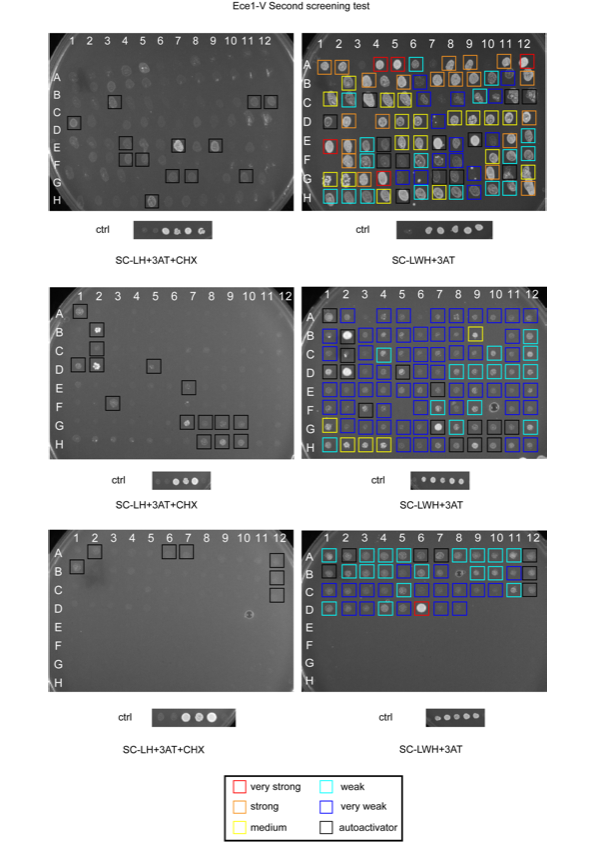

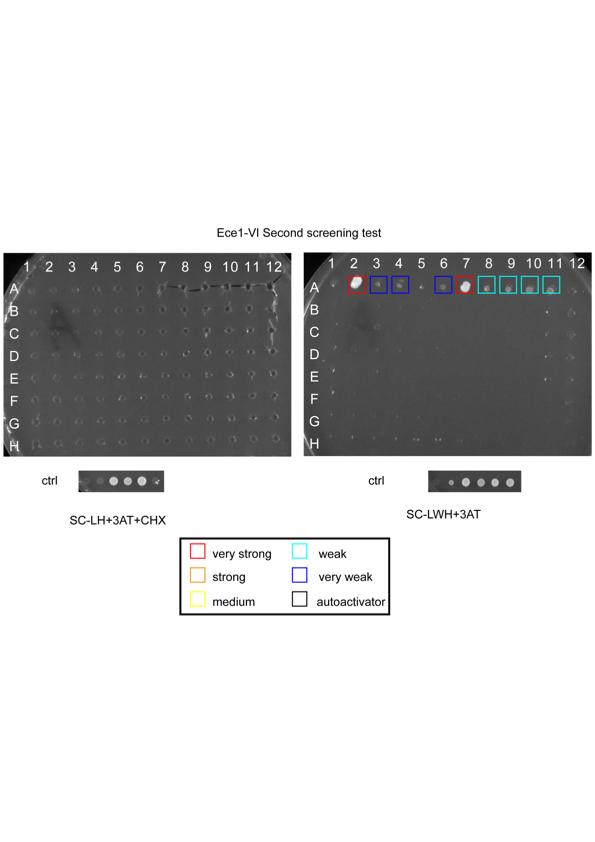

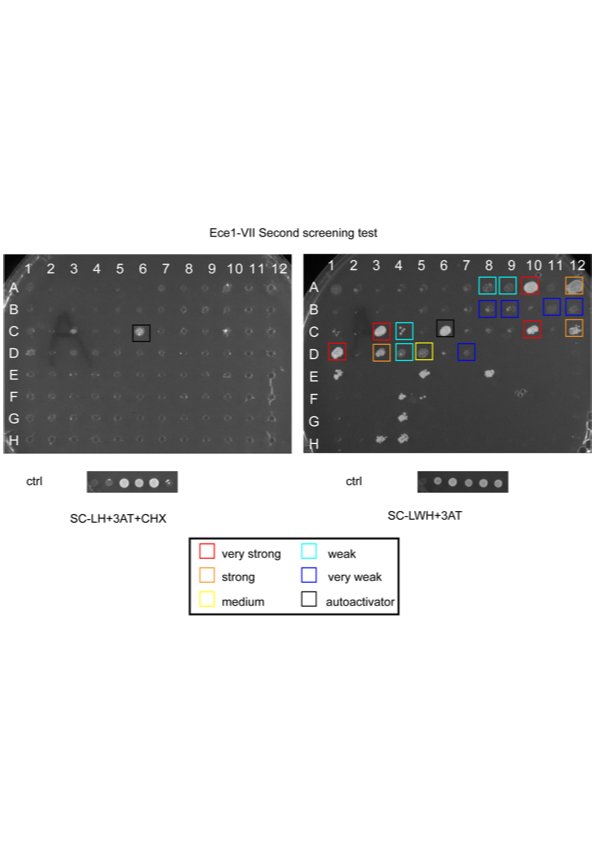

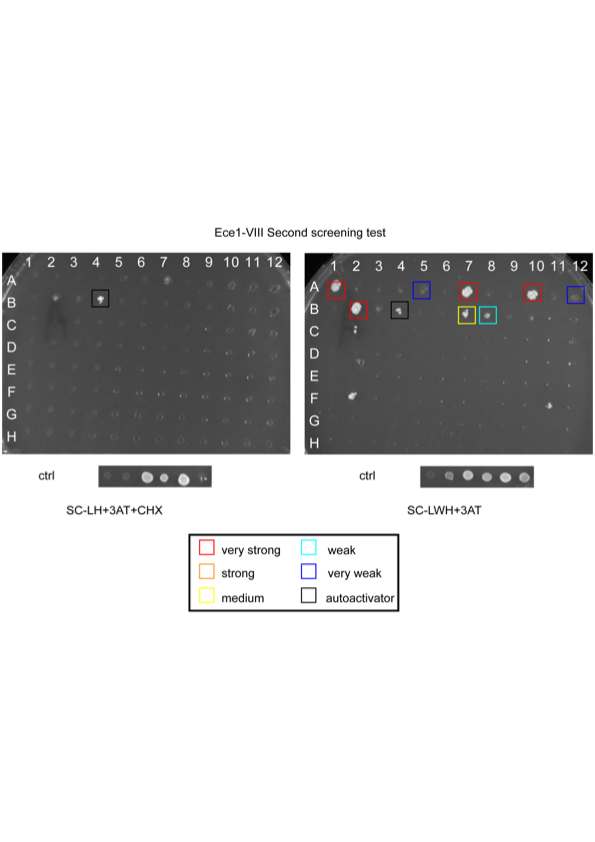

Supplement: Supplementary file 9 — Source Data [file 41467_2024_46141_MOESM9_ESM.zip › Source_Data/Source Data of Fig. 1.docx]
