# Supplementary material for: Global fungal-host interactome mapping identifies host targets of candidalysin
Source: Nat Commun. 2024 Feb 27;15:1757. doi: 10.1038/s41467-024-46141-x (PMC10899660; doi:10.1038/s41467-024-46141-x)

**Source Data: Uncropped scans of source gels used in this study**

**Fig. 4g**

CCNH
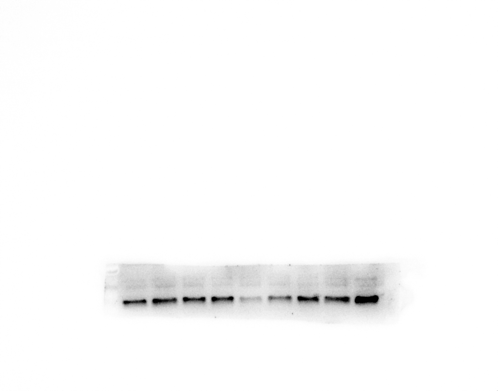


γ-H2AX
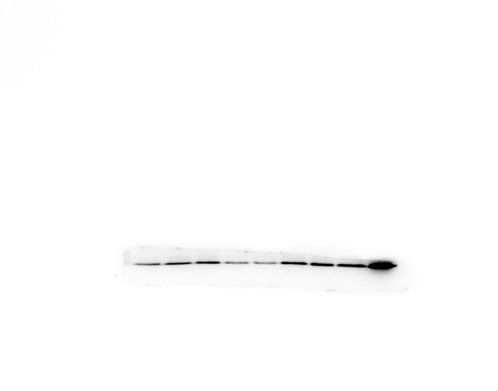


GAPDH
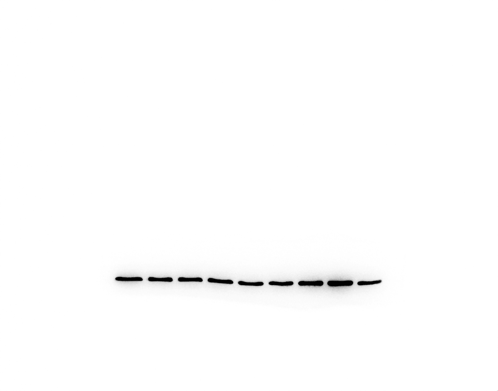


**Fig. 4h**

CCNH
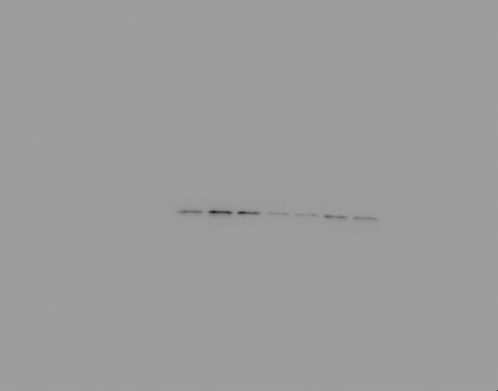


γ-H2AX
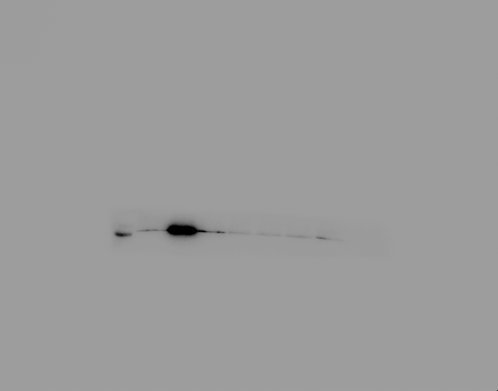


GAPDH
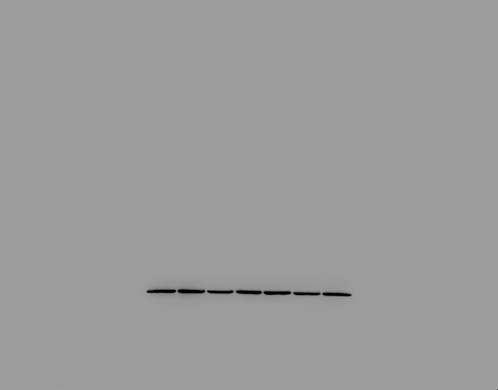


**Fig. 4i**

Anti-Flag **
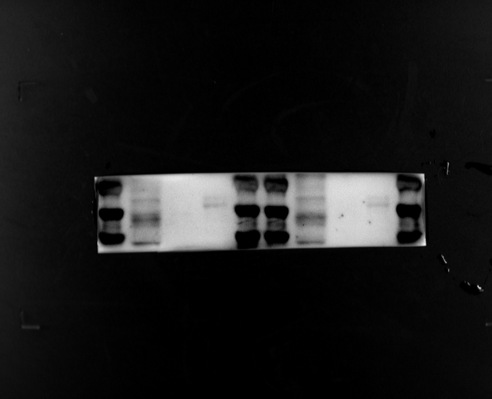
**

Anti-GFP **
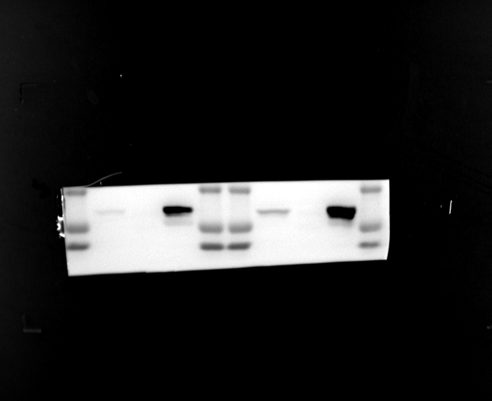
**

**Fig. 4n**

CCNH **
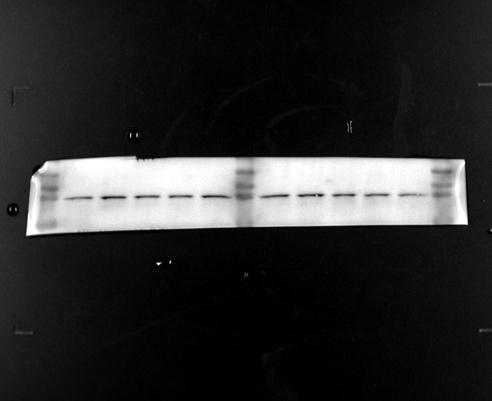
**

γ-H2AX
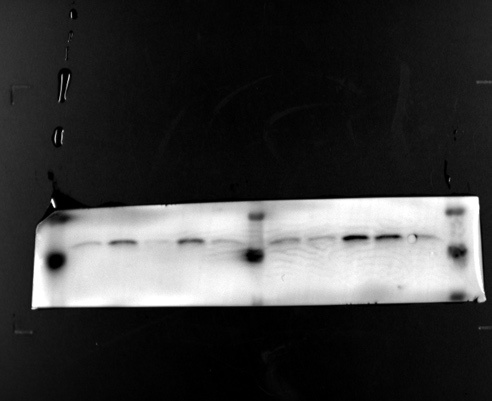


p-CDK1/2
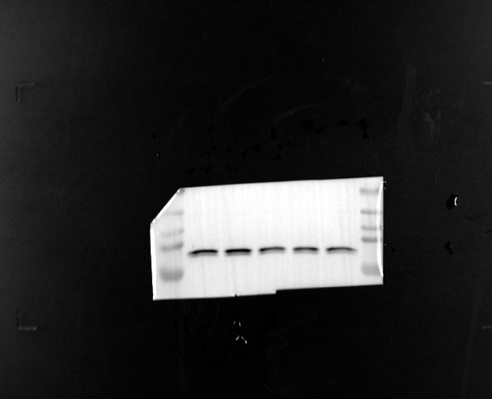


GAPDH
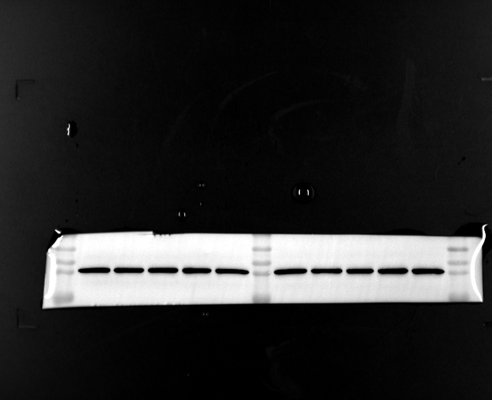

Supplement: Supplementary file 9 — Source Data [file 41467_2024_46141_MOESM9_ESM.zip › Source_Data/Source Data of Uncropped Scans of Western Blot Gels.docx]
