# Supplementary figures and images for: Global fungal-host interactome mapping identifies host targets of candidalysin
Source: Nat Commun. 2024 Feb 27;15:1757. doi: 10.1038/s41467-024-46141-x (PMC10899660; doi:10.1038/s41467-024-46141-x)

**The data analysis procedure of flow cytometry, related to Fig. 4b.**

**
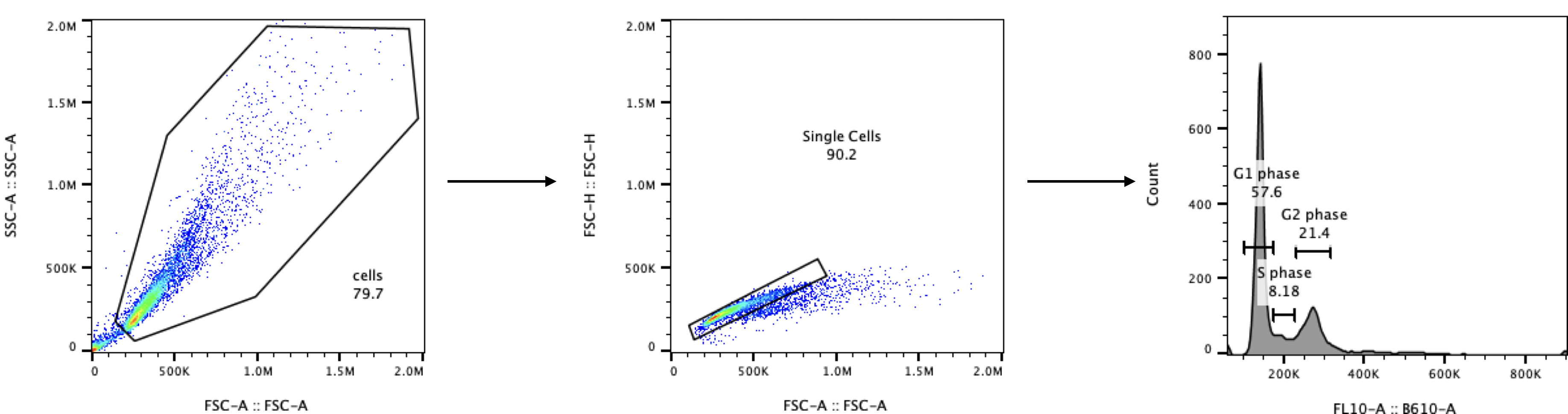
**

Supplement: Supplementary file 9 — Source Data [file 41467_2024_46141_MOESM9_ESM.zip › Source_Data/Source Data of Flow Cytometry Analysis.docx]
